# Novel PPARα agonist MHY553 alleviates hepatic steatosis by increasing fatty acid oxidation and decreasing inflammation during aging

**DOI:** 10.18632/oncotarget.17695

**Published:** 2017-05-08

**Authors:** Seong Min Kim, Bonggi Lee, Hye Jin An, Dae Hyun Kim, Kyung Chul Park, Sang-Gyun Noh, Ki Wung Chung, Eun Kyeong Lee, Kyung Mok Kim, Do Hyun Kim, Su Jeong Kim, Pusoon Chun, Ho Jeong Lee, Hyung Ryong Moon, Hae Young Chung

**Affiliations:** ^1^ Molecular Inflammation Research Center for Aging Intervention (MRCA), College of Pharmacy, Pusan National University, Busan 46241, Republic of Korea; ^2^ Research Institute of Life Science and College of Veterinary Medicine, Gyeongsang National University, Jinju 52828, Republic of Korea; ^3^ Korean Medicine (KM)-Application Center, Korea Institute of Oriental Medicine (KIOM), Daegu 41062, Republic of Korea; ^4^ College of Pharmacy, Inje University, Gimhae 621-749, Republic of Korea

**Keywords:** MHY553, PPARα agonist, hepatic steatosis, fatty acid oxidation, aging

## Abstract

Hepatic steatosis is frequently observed in obese and aged individuals. Because hepatic steatosis is closely associated with metabolic syndromes, including insulin resistance, dyslipidemia, and inflammation, numerous efforts have been made to develop compounds that ameliorate it. Here, a novel peroxisome proliferator-activated receptor (PPAR) α agonist, 4-(benzo[d]thiazol-2-yl)benzene-1,3-diol (MHY553) was developed, and investigated its beneficial effects on hepatic steatosis using young and old Sprague-Dawley rats and HepG2 cells.

Docking simulation and Western blotting confirmed that the activity of PPARα, but not that of the other PPAR subtypes, was increased by MHY553 treatment. When administered orally, MHY553 markedly ameliorated aging-induced hepatic steatosis without changes in body weight and serum levels of liver injury markers. Consistent with *in vivo* results, MHY553 inhibited triglyceride accumulation induced by a liver X receptor agonist in HepG2 cells. Regarding underlying mechanisms, MHY553 stimulated PPARα translocation into the nucleus and increased mRNA levels of its downstream genes related to fatty acid oxidation, including CPT-1A and ACOX1, without apparent change in lipogenesis signaling. Furthermore, MHY553 significantly suppresses inflammatory mRNA expression in old rats. In conclusion, MHY553 is a novel PPARα agonist that improved aged-induced hepatic steatosis, in part by increasing β-oxidation signaling and decreasing inflammation in the liver. MHY553 is a potential pharmaceutical agent for treating hepatic steatosis in aging.

## INTRODUCTION

Aging is often preceded by changes in biochemical predictors, including oxidative injury, increases in lipid peroxides, and DNA oxidative damage [1]. The process of aging results in a progressive decrease in normal cellular functions. Accordingly, the aged liver may be more sensitive to drugs and toxins, thereby having decreased regenerative capacity [2]. The percentage of death caused by liver diseases notably increases in humans over the age of 45 years.

Non-alcoholic fatty liver disease (NAFLD) is a pathological change characterized by the accumulation of triglycerides in hepatocytes [3]. NAFLD is related to aging-related metabolic syndromes, such as insulin resistance, dyslipidemia, and inflammation [4]. In the presence of a fatty liver, the hyperinsulinemic condition fails to inhibit free fatty acid flux in adipose tissues, causing free fatty acid to be absorbed by the liver [5]. This state drives triglyceride accumulation and inflammation in the liver when the lipid-storing functions of adipose tissues are overwhelmed. Therefore, NAFLD is a multi-factorial disease in which tissue to tissue communication is impaired.

The peroxisome proliferator-activated receptors (PPARs) belong to the nuclear receptor superfamily and are well-known ligand-activated transcription factors [6]. The PPAR family includes PPARα, PPARβ, and PPARγ. Although the tissue expression pattern of the various PPARs is different, together they regulate a variety of cellular processes related to energy metabolism, including gluconeogenesis, lipid synthesis, lipid uptake, and lipid breakdown [7]. In the liver, PPARα is highly expressed and its activation stimulates the expression of genes related to peroxisomal β-oxidation, such as peroxisomal acyl-coenzyme A oxidase (ACOX1) [8]. In addition, PPARα controls the critical reactions of mitochondrial β-oxidation, by increasing carnitine palmitoyltransferase 1 (CPT-1) expression levels [9]. In addition, PPARα inhibits lipogenesis in the liver by both direct and indirect mechanisms. PPARα was reported to indirectly modulate SREBP-1c transcription through cross-control with liver X receptor (LXR) signaling [10]. PPARα is necessary for the LXRα-dependent response of stearoyl-CoA desaturase-1 (SCD-1) and fatty acid synthase (FAS) to insulin in re-fed mice, indicating a possible role for PPARα in the endogenous ligand synthesis of LXRα [11]. Furthermore, PPARα activation suppresses inflammatory signaling pathways through the inhibition of cytokine-mediated IL-6 gene expression via interference with the activator protein-1 (AP-1). The PPARα-induced trans-repression includes direct physical interactions between PPARα and the c-Jun N-terminal kinase (JNK)-responsive part of c-Jun in combination with c-Fos, which forms the AP-1 early response transcription factor [12].

PPARβ is ubiquitously expressed and its functions are similar to PPARα in that it increases fatty acid oxidation signaling in metabolic organs such as the liver, skeletal muscle, adipose tissue, and heart [13]. Otherwise, PPARγ appears to stimulate fatty liver development.

In the current study, we investigated whether MHY553 is a new PPARα agonist, docking simulation was performed followed by *in vitro* and *in vivo* studies. Our data showed that MHY553 can bind to and activate PPARα, contributing to the amelioration of hepatic steatosis and inflammation associated with aging. Our study suggests mhy553 may be a new pharmaceutical compound that can intervene aging-associated with metabolic syndrome.

## RESULTS

### MHY553 as a potential PPARα agonist

**Figure 1:**
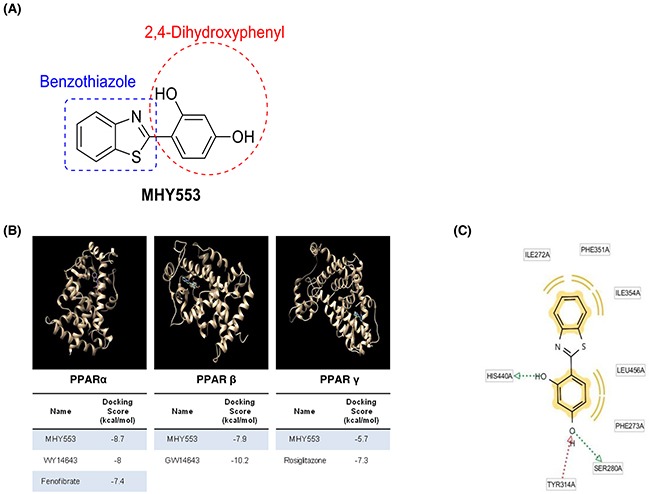
MHY553 can directly bind to PPARα **(A)** Structure of MHY553. **(B)** Computational structure prediction for docking simulation between each of the three PPAR subtypes and MHY553. The gray zone indicates the active site of PPAR subtypes (PDB ID: PPARα, 1K7L; PPARβ/δ, 1GWX; and PPARγ, 3DZY), magenta indicates MHY553, and cyan indicates the respective positive control for each of the three PPAR subtypes. The tables indicate the docking scores of MHY553 and each positive control with three PPAR subtypes. **(C)** The PPARα binding sites of MHY553 are shown. Yellow indicates the hydrophobic interaction, green indicates the aromatic interaction, and red indicates the hydrogen bond.

A number of compounds among the benzothiazole family were identified as PPARα agonists (unpublished data). We investigated the binding affinity of these compounds to PPARα, the AutoDock Vina docking simulation program was used. Among them, MHY553 (Figure 1A) had a relatively high binding affinity for PPARα. In addition, the docking simulation indicated that the binding affinity of MHY553 for PPARα was greater than that of well-known PPARα agonists, including WY14643 and fenofibrate (Figure 1B). The binding energy of MHY553 to PPARα was -8.7 kcal/mol and the binding energies of WY14643 and fenofibrate to PPARα were -8.0 and -7.4 kcal/mol, respectively. To investigate the binding residue analysis, LigandScout 3.0 software was used. The data indicated that the Ser280 and His440 residues of PPARα were involved in hydrogen bonding with MHY553 as hydrogen acceptors and the Tyr314 residue of PPARα was found to bind with MHY553 as a hydrogen donor (Figure 1C). Therefore, these residues functioned as important determinants of activator activity and total binding affinity. These results suggested that MHY553 was a potential PPARα agonist.

### MHY553 increased the transcriptional activity of PPARα

**Figure 2:**
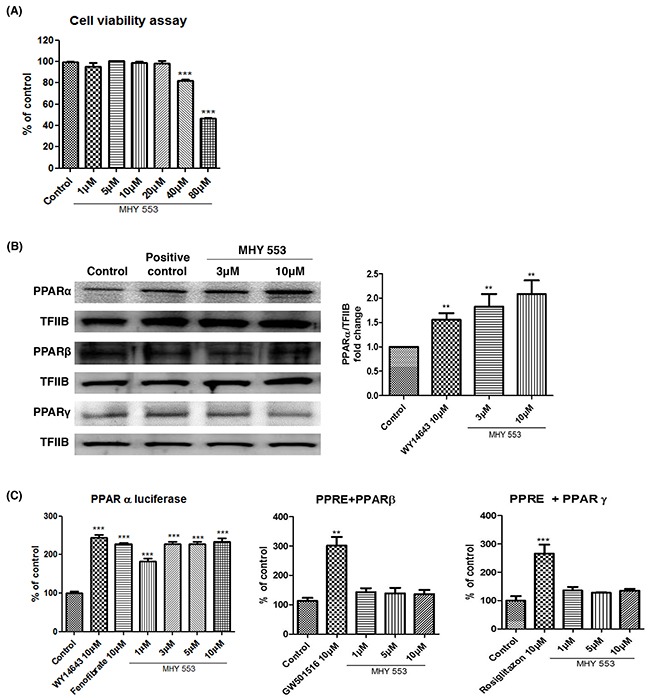
MHY553 increases transcriptional activity of PPARα without cell cytotoxicity **(A)** HepG2 cells were treated with various concentrations of MHY553 for 24 h, then an cytotoxity assay was performed (n = 8). **(B)** The nuclear levels of PPARs in HepG2 cells treated with MHY553 for 90 min were analyzed by Western blotting. The respective PPAR agonists (PPARα; WY14643 10 μM, PPARβ; GW14643 10 μM or PPARγ; rosiglitazone 10 μM) were treated (n = 4). Transcription factor II B (TFIIB) was used as the loading control. **(C)** For luciferase, the 3X-PPRE-TK-LUC plasmid and respective PPARα, PPARβ and PPARγ expression vectors were transfected into HepG2 cells. Twenty-four hours after the transfection, the cells were treated with the indicated compounds for 5 h. A t-test was used to determine the significance of the differences. ^*^p < 0.01 *vs*. Control; ^**^*p < 0.001 *vs*. Control.

We evaluated whether MHY553 was cytotoxic, 3-(4,5-dimethylthiazol-2-yl)-2,5-diphenyltetrazolium bromide (MTT) assay was performed using HepG2 cells. MHY553 did not affect cell viability at concentrations up to 20 μM over 24 h (Figure 2A). Upon ligand binding, PPARs translocate to the nucleus to induce the transcription of target genes. Western blotting indicated that the nuclear distribution of PPARα was higher in the MHY553-treated groups. In addition, the effect was stronger than that observed with WY14643 treatment. However, MHY553 did not affect the nuclear distribution of other PPAR subtypes, such as PPARβ and PPARγ, in HepG2 cells (Figure 2B).

To verify that MHY553 induced the transcriptional activity of PPARα, a luciferase assay was performed in HepG2 cells. The luciferase assay indicated that the transcriptional activity of PPARα was increased by MHY553 treatment and the effect was comparable to that of WY14643 and fenofibrate treatment. However, MHY553 did not affect the transcriptional activity of PPARβ and PPARγ, in HepG2 cells (Figure 2C). These results confirmed that MHY553 was a strong PPARα agonist.

### MHY553 improved hepatic steatosis in old rats

**Figure 3:**
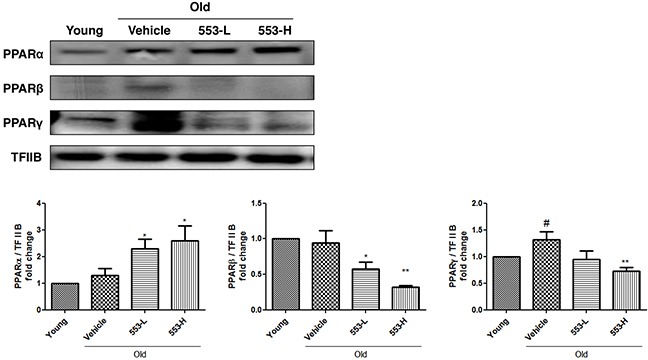
MHY553 increases PPARα translocation to the nucleus in the *in vivo* model The nuclear levels of three PPAR subtypes in the liver of old rats orally treated with MHY553 for 30 days were analyzed by Western blotting. TFIIB was used as the loading control. The blots were quantified by densitometry (n = 6). Young (6 month old) rats: Young; Old (21 month old) rats, Old; Vehicle; MHY553 (3 mg/kg·day)-treated old rats, 553-L; MHY553 (5 mg/kg·day)-treated old rats, 553-H. A t-test was used to determine the significance of the differences. ^#^*p* < 0.05 *vs*. Young; *p < 0.05 *vs*. Vehicle; ^*^p < 0.01 *vs*. Vehicle.

We first verified that MHY553 activated PPARα in the rat liver, Western blotting was performed. Consistent with the effects of MHY553 on PPARα activation in HepG2 cells, MHY553 increased the translocation of PPARα into the nucleus. In contrast, protein levels of PPARβ and PPARγ in the nucleus were not increased by MHY553 treatment. These results suggested that MHY553 was a PPARα specific agonist (Figure 3).

**Figure 4:**
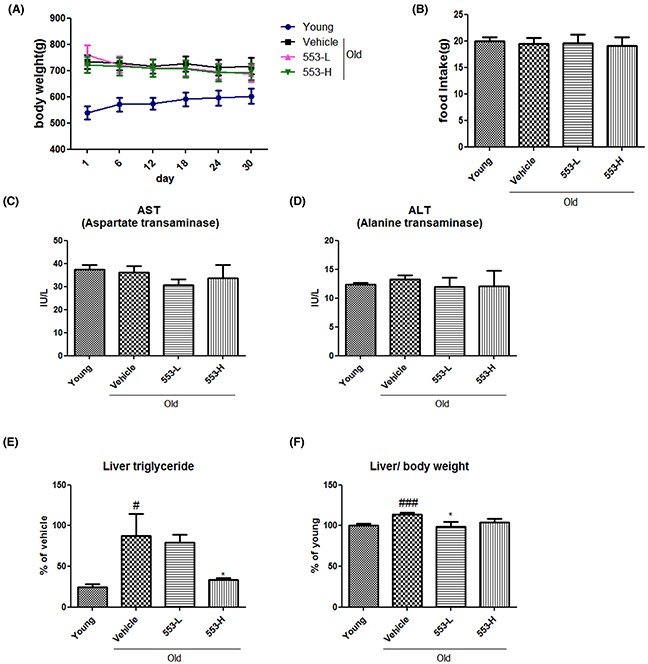
MHY553 ameliorated aging-induced hepatic steatosis without changes in body weight and serum levels of liver injury markers in rats MHY553 (3 mg/kg·day) or (5 mg/kg·day) was orally administered for 30 days in old rats. Body weight and food intake were measured once every six days. **(A)** Body weight. **(B)** Food intake. **(C)** Serum aspartate aminotransferase (AST). **(D)** Alanine aminotransferase (ALT). **(E)** Liver triglycerides. **(F)** Liver weight per body weight. Young (6 month old) rats: Young; Old (21 month old) rats, Old; Vehicle; MHY553 (3 mg/kg·day)-treated old rats, 553-L; MHY553 (5 mg/kg·day)-treated old rats, 553-H. A t-test was used to determine the significance of the differences. ^##^*p* < 0.01 *vs*. Young; ^###^*p* < 0.001 *vs*. Young; *p < 0.05 *vs*. Vehicle.

To investigate the beneficial effects of MHY553 *in vivo*, MHY553 was orally administered at the concentrations of 3 mg/(kg·day) or 5 mg/(kg·day) for 30 days in old rats. Body weight and food intake were not different between the groups during the study (Figure 4A and 4B), indicating that MHY553 did not affect energy balance. To test whether MHY553 had hepatotoxic effects *in vivo*, liver injury markers, including aspartate aminotransferase (AST) and alanine aminotransferase (ALT), were measured in serum. The results indicated that MHY553 treatment did not affect the levels of these markers (Figure 4C and 4D). In addition, MHY553 did not affect serum triglyceride, total cholesterol, serum non-esterified fatty acids (NEFA), or glucose (Supplementary Figure 1A-1D). Hepatic steatosis is commonly found during aging together with other metabolic diseases. We examined the effect of MHY553 on the fatty liver, hepatic triglyceride levels were measured in rats. MHY553 attenuated aging-induced hepatic steatosis as evidenced by a significant decrease in hepatic triglyceride proportion (Figure 4E). As expected, the liver weight per body weight of the old groups was elevated compared to the young groups, whereas MHY553 treatment appeared to decrease the liver weight per body weight (Figure 4F). These data indicated that MHY553 alleviated aging-induced hepatic steatosis.

### MHY553 increased fatty acid oxidation in old rats

**Figure 5:**
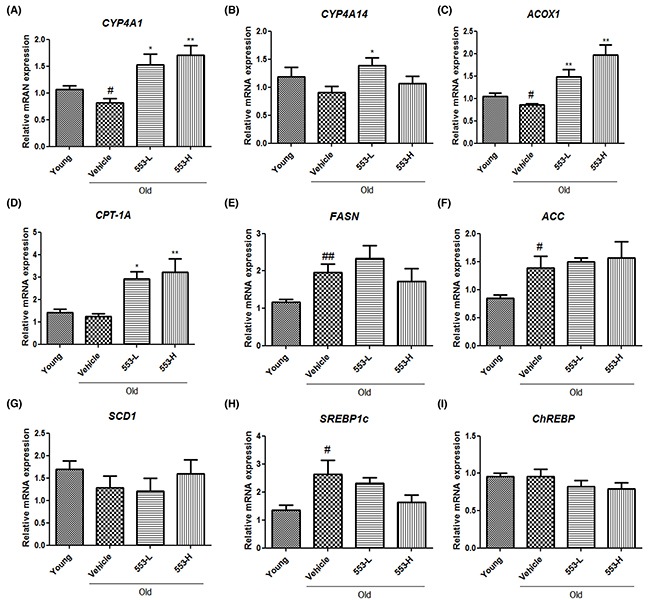
MHY553 increases mRNA levels of fatty acid oxidation-related genes, but mRNA levels of lipogenesis-related genes are not changed by MHY553 in old rat livers mRNA levels of **(A)** CYP4A1, **(B)** CYP4A14, **(C)** ACOX1, **(D)** CPT-1A, **(E)** FASN, **(F)** ACC, **(G)** SCD1, **(H)** SREBP1c, and **(I)** ChREBP in liver tissue were analyzed by qRT–PCR. Young (6 month old) rats, Young; Old (21 month old) rats, Old; Vehicle; MHY553 (3 mg/kg·day)-treated old rats, 553-L; MHY553 (5 mg/kg·day)-treated old rats, 553-H. A t-test was used to determine the significance of the differences. ^#^*p* < 0.05 *vs*. Young; ^##^*p* < 0.01 *vs*. Young; *p < 0.05 *vs*. Vehicle; ^*^p < 0.01 *vs*. Vehicle.

As a potential factor underlying the MHY553-mediated decrease in the hepatic triglyceride level, fatty acid oxidation signaling increased by PPARα was investigated using qPCR. The mRNA levels of CYP4A1 were decreased in the old groups and dose-dependently increased in the MHY553-treated groups (Figure 5A). The mRNA levels of CYP4A14 also were increased by MHY553 (3 mg/(kg·day)) treatment (Figure 5B). Furthermore, MHY553 significantly increased the mRNA expression of CPT-1A and ACOX1 (Figure 5C and 5D). These qPCR data suggested that MHY553 induced fatty acid oxidation, contributing to improvement of hepatic steatosis.

### MHY553 did not effect on *de novo* lipid synthesis in old rats

As a potential factor underlying the MHY553-mediated decrease in the hepatic triglyceride level, *de novo* lipid synthesis-related genes, including FASN, ACC, SCD1, SREBP1c and ChREBP, were examined. These genes were mostly increased in the old groups compared to the young groups (Figure 5E-5I). However, MHY553 did not change the expression of these genes, indicating that MHY553 did not significantly affect lipogenesis signaling. These data indicated that MHY553 improved hepatic steatosis, possibly by elevating fatty acid oxidation without an apparent change in lipogenesis signaling.

### Effects of MHY553 on inflammation in old rats

**Figure 6:**
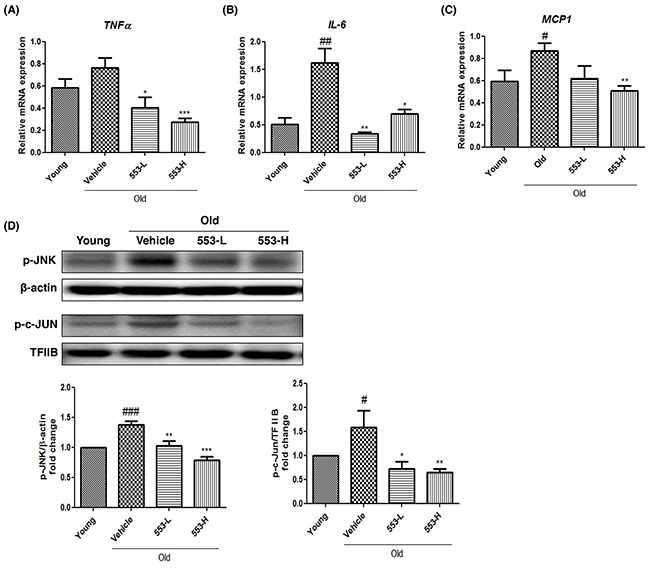
MHY553 attenuates aging-induced inflammation in liver tissue Pro-inflammatory cytokines including **(A)** TNFα, **(B)** IL-6, and **(C)** MCP1 were analyzed by qRT–PCR. **(D)** Protein levels of phospho c-Jun and phospho JNK were analyzed by Western blot analysis. TFIIB in nuclear and β-actin in cytosol were used as a loading control. Graphs represent a densitometric analysis of Western blots. Young (6 month old) rats, Young; Old (21 month old) rats, Old; Vehicle; MHY553 (3 mg/kg·day)-treated old rats, 553-L; MHY553 (5 mg/kg·day)-treated old rats, 553-H. A t-test was used to determine the significance of the differences. ^#^*p* < 0.05 *vs*. Young; ^##^*p* < 0.01 *vs*. Young; ^###^*p* < 0.001 *vs*. Young; *p < 0.05 *vs*. Vehicle; ^*^p < 0.01 *vs*. Vehicle; ^**^*p < 0.001 *vs*. Vehicle.

Because fatty liver may be accompanied by hepatic inflammation and PPARα regulates inflammation, the effects of MHY553 on inflammation were investigated. The mRNA levels of pro-inflammatory cytokines, including TNFα, MCP1, and IL-6, were increased in the old groups compared with the young groups, but were significantly decreased by MHY553 treatment (Figure 6A-6C). Because AP-1 and NF-κB are key regulators of pro-inflammatory cytokines, the nuclear protein levels of p-c-JUN and p-p65 was evaluated by Western blotting. As expected, the protein expression of p-c-JUN, but not that of p-p65 (data not shown), was increased in the old groups compared to the young groups. Interestingly, MHY553 reduced p-c-JUN expression, but not p-p65 expression. In addition, upstream of c-JUN, p-JNK expression was decreased in MHY553-treated old rats (Figure 6D). These results indicated that MHY553 played an anti-inflammatory role in the old rat liver.

### MHY553 induced fatty acid oxidation and reduced triglyceride accumulation in HepG2 cells

**Figure 7:**
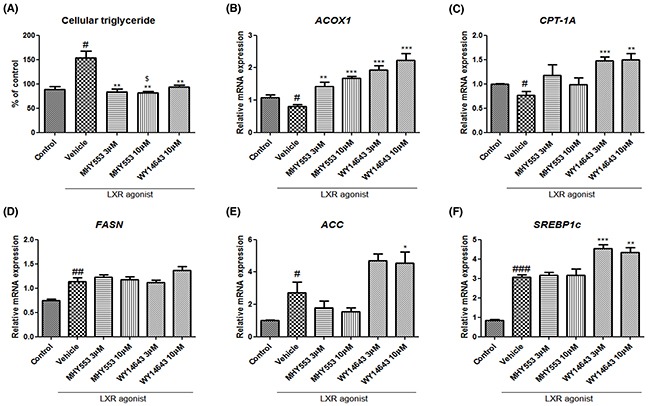
MHY553 inhibits triglyceride accumulation induced by a liver X receptor agonist (T0901317) through fatty acid oxidation in HepG2 cells HepG2 cells were pretreated with vehicle (DMSO), 3 μM MHY553, 10 μM MHY553, or 10 μM WY14643 for 1 h and then treated with 1 μM LXR agonist (T0901317) for 48 h. **(A)** Cellular triglyceride, **(B)** ACOX1, **(C)** CPT-1A, **(D)** FASN, **(E)** ACC, and **(F)** SREBP1c. A t-test was used to determine the significance of the differences. ^#^*p* < 0.05 *vs*. Control; ^##^*p* < 0.01 *vs*. Control; ^###^*p* < 0.001 *vs*. Control; *p < 0.05 *vs*. Vehicle; ^*^p < 0.01 *vs*. Vehicle; ^**^*p < 0.001. *vs*. Vehicle; ^$^*p* < 0.05 *vs*. WY14643 10 μM of (A) data.

To determine the direct effect of MHY553 on the reduction of hepatic triglyceride levels, MHY553 pre-treated HepG2 cells were treated with a well-known LXR agonist, T0901317. LXRs have been characterized as major regulators of hepatic fatty acid biosynthesis through the induction of SREBP1c, ACC1, FASN, and SCD1. As expected, the LXR agonist increased hepatic triglyceride levels (Figure 7A). In addition, this agonist decreased fatty acid oxdiation-related genes, such as ACOX1 and CPT-1A (Figure 7B and 7C), and increased lipogenesis-related genes, including FASN, ACC, and SREBP1c (Figure 7D-7F).

Our data showed that MHY553 decreased triglyceride accumulation induced by the LXR agonist in HepG2 cells (Figure 7A). As indicated in the *in vivo* study, old rat fatty acid oxidation and lipogenesis-related genes were examined as a potential factor underlying the MHY553-mediated decrease in the hepatic triglyceride level. MHY553 dose-dependently increased CPT-1A and ACOX1, both of which are associated with fatty acid oxidation (Figure 7B and 7C). The mRNA expression of these genes in WY14643-treated groups was higher than that in the MHY553-treated groups. However, MHY553 did not increase FASN, ACC, and SREBP1c, which are associated with lipogenesis (Figure 7D-7F). These data suggested that MHY553 directly increased fatty acid oxidation signaling in HepG2 cells, contributing to the reduction of hepatic triglyceride levels.

## DISCUSSION

In the present study, MHY553 was found to be a PPARα agonist based on an *in silico* protein docking simulation, Western blotting, and a PPRE-luciferase assay. Oral administration of MHY553 in old rats substantially alleviated aging-induced hepatic steatosis without changes in body weight or serum levels of liver injury markers. In the liver, MHY553 greatly elevated the mRNA expression of fatty acid oxidation-related genes. To evaluate whether MHY553 had a direct effect on the liver, lipid accumulation was induced in HepG2 cells by treatment with the LXR agonist, T0901317, which is a well-known stimulator of hepatic steatosis through the induction of *de novo* lipogenesis [14]. MHY553 reduced triglyceride accumulation induced by T0901317 more than WY14643 in HepG2 cells.

The major aspects of lipid metabolism involve fatty acid oxidation to produce energy and lipid synthesis, which is called lipogenesis. A number of studies have indicated that fatty acid oxidation signaling is down-regulated [15] and lipogenesis signaling is up-regulated during the aging process [16]. As expected, fatty acid oxidation-related genes, such as ACOX1 and CPT-1A, were reduced and lipogenesis-related genes, including SREBP1, FASN, and ACC, were increased in old rats. Both WY14643 and MHY553 greatly increased fatty oxidation-related gene expression. However, WY14643 increased lipogenesis-related genes in LXR agonist-treated HepG2 cells, as reported in other studies [17], although the mechanisms are unclear. On the other hand, MHY553 did not affect lipogenesis-related genes, including SREBP1c, FASN and ACC, indicating that increased fatty acid oxidation contributed to the MHY553-mediated amelioration of hepatic lipid accumulation. In addition, MHY553 may be better than WY14643 in that it did not enforce the lipogenesis pathway. Further *in vivo* studies are necessary to investigate whether MHY553 is more effective than WY14643 against fatty liver.

Fatty liver frequently involves an increase in inflammatory signaling, which is closely related to hepatic insulin resistance, fibrosis, and apoptosis [18]. The anti-inflammatory effect of MHY553 was also observed in the liver of old rats. Although the mechanisms are unclear, it is likely that MHY553 decreased hepatic inflammation directly by activation of PPARα or indirectly by reducing lipid accumulation. A number of studies indicated that hepatic PPARα activation was associated with a reduction in inflammatory gene expression [19]. In addition, PPARα activation appeared to inhibit AP-1 signaling [13]. Consistent with this, MHY553 suppressed aging-induced AP-1 and JNK activation, both of which are key players in hepatic inflammation. On the other hand, it is also likely that MHY553 inhibited hepatic inflammation by decreasing triglyceride accumulation because fatty acids and other lipid-derivatives possibly produced from triglyceride breakdown have been shown to activate hepatic inflammatory signaling and induce lipotoxicity [20]. Therefore, MHY553 appeared to suppress hepatic inflammation by both a direct and an indirect effect of PPARα activation.

In summary, PPAR agonists have various beneficial effects that alleviate insulin resistance, endoplasmic reticulum stress, and inflammation [21]. MHY553 improved hepatic steatosis through fatty acid oxidation signaling and decreased inflammation during aging. Therefore, MHY553 is a potential pharmaceutical agent that may ameliorate metabolic syndrome derived from aging.

## MATERIALS AND METHODS

### Animal experiments

To estimate the effects of MHY553 on hepatic steatosis and inflammation in aging, male, young (6 months) and old (21 months), Sprague-Dawley (SD) rats were purchased from Samtako (Kyoung-Ki, South Korea) and acclimated to the animal care facility for one week. Rats were housed in a 12 h light/dark cycle at 23 ± 1°C and 50 ± 5% relative humidity with free access to a standard rodent chow (Samtako) and water. Six-month-old ad libitum fed rats were used as young controls (n=6). MHY553 (3 mg/(kg·day) or 5 mg/(kg·day)) was orally administered for 30 days in old rats. Body weight and food intake was measured once every six days. Livers were collected for biochemical analysis and Western blotting. The animal protocol was approved by the Pusan National University Institutional Animal Care and Use Committee (PNU-IACUC) with respect to ethical procedures and scientific care (PNU-2017-1428).

### *In silico* protein-ligand docking simulation

The crystal structures of human PPARα, PPARβ, and PPARγ were obtained from the Protein Data Bank (PDB) archives (entry code PPARα: 1K7L, PPARβ: 1GWX, PPARγ: 3DZY) and used as targets for docking simulation. The AutoDock Vina program and the tool's manual were used. To define the docking pockets of the PPAR subtypes, a set of predefined active sites in human PPARs were used. Docking simulations were performed between PPAR subtypes and MHY553. To prepare compounds for the docking simulation, 2D structures were first converted to 3D structures. Charges were then calculated and hydrogen atoms were added using the ChemOffice program (http://www.cambridgesoft.com).

### Cell culture

HepG2 cells, a line of human hepatoma cells, were obtained from America Type Culture Collection (Manassas, VA, USA). The cells were grown in Dulbecco's modified eagle medium (DMEM, Welgene, Gyeongsan, Gyeongsangbuk-do, Korea) with 10% fetal bovine serum (FBS; Welgene) containing 100 U/mL penicillin and 100 μg/mL streptomycin (Welgene) at 37°C in a 5% CO_2_ atmosphere. The medium was replaced with fresh medium after one day to remove non-adherent cells or cell debris.

### Cell viability

A cell viability assay was carried out using 3-[4,5-dimethylthiazol-2-yl]-2,5-diphenyltetrazolium bromide (EZ-CYTOX, Dogen, Seoul, Korea). HepG2 cells (2 × 10^4^) were plated in each well of a 96-well cell culture plate and incubated overnight. After the cells were treated with MHY553 at concentrations ranging from 1 to 80 μM for 24 h, the EZ-CYTOX reagent was added to each well (10 μL per 100 μL media) and the plate was incubated for 1 h. Absorbance in each well was determined at 560 nm using a microplate reader.

### Luciferase assay

For the peroxisome proliferator response element (PPRE)-driven luciferase assay, 1.5 × 10^4^ HepG2 cells per well were cultured in a 96-well cell culture plate in 100 μL of DMEM supplemented with 10% FBS. The PPRE-X3-TK-LUC plasmid (0.1 μg) (Dr. Christoper K. Glass, University of California, San Diego, CA, USA) and 0.01 μg PPARα expression vector (Dr. Han Geuk Seo, Konkuk University, Seoul, South Korea) were transfected with 0.1 μL of Lipofectamine 3000 reagent and 0.2 μL P3000 (Invitrogen, Carlsbad, CA, USA) complexes in Opti-MEM (Invitrogen), according to the manufacturer's instructions. After 24 h of transfection, cells were treated with MHY553, WY14643, or fenofibrate for 5 h. Luciferase activity was measured using the One-Glo Luciferase Assay System (Promega, Madison, WI, USA) and a luminescence plate reader (Berthold Technologies GmbH & Co., Bad Wildbad, Germany).

### Protein extraction and immunoblot analysis

To extract the cytosol and nuclear fractions from cells and tissues, the cell pellet or tissue was homogenized in 10 mM Tris buffer (pH 8.0), 20 mM NaF, 2 mM sodium orthovanadate, 20 mM β-glycerophosphate, 0.01 mM dithiothreitol (DTT), 1 mM ethylenediaminetetraacetic acid (EDTA), 0.5 mM phenylmethylsulfonyl fluoride (PMSF), 0.1% Nonidet P-40 (NP-40), and protease inhibitor. It was then kept on ice for 20 min and centrifuged at 12,000 rpm and 4°C for 10 min. The supernatants were used as the cytosol fraction. The pellets were washed three times with cytosol lysis buffer. To extract the nuclear fraction, the pellet was homogenized and suspended in 10 mM Tris buffer (pH 8.0), 100 mM NaCl, 50 mM KCl, 10% (v/v) glycerol, 0.01 mM DTT, 0.1 mM EDTA, 20 mM β-glycerophosphate, 2 mM sodium orthovanadate, 20 mM NaF, 1 mM EDTA, 0.5 mM PMSF, and protease inhibitor. It was then incubated on ice for 30 min and centrifuged at 12,000 rpm and 4°C for 10 min. The resultant supernatants were used as the nuclear proteins and stored at -80°C. The protein concentration was measured using bicinchoninic acid (BCA; Thermo Scientific, Waltham, MA, USA) and bovine serum albumin (BSA) was used as the standard. Samples were prepared in a gel buffer [12.5 mM Tris buffer (pH 6.8), 4% sodium dodecyl sulfate, 20% glycerol, 10% 2-mercaptoethanol, and 0.2% bromophenol blue] and kept at 100°C for 5 min. Sodium dodecyl sulfate-polyacrylamide gel electrophoresis (SDS-PAGE) containing 6% to 17% acrylamide was used to separate equal concentrations of protein. Using a wet transfer system, the gel was transferred to polyvinylidene difluoride (PVDF) membranes at 90V for 90 min. The membranes were immediately located in blocking buffer [10 mM Tris buffer (pH 7.5), 100 mM NaCl, 0.1% Tween 20, and 5% non-fat milk]. Blotting was done at 25°C for 30 min and then membranes were incubated with specific primary antibody at 4°C for 16 h. Secondary antibody was then added followed by a horseradish peroxidase-conjugated anti-rabbit antibody (Santa Cruz, 1:10,000), anti-goat antibody (Santa Cruz, 1:10,000), or anti-mouse antibody (Santa Cruz, 1:10,000) at 25°C for 1 h. Antibody labeling was used to detect antibodies with WesternBright^TM^ ECL reagent (Advansta, Menlo Park, CA, USA).

### Isolation and quantification of RNA

Tissue RNA was homogenized in the presence of RiboEx (GeneAll, Seoul, Korea) using a bead homogenizer (TissueLyser, Qiagen, Hilden, German) according to the manufacturer's instructions. A cell pellet was purified in the presence of RiboEx without the bead homogenizer. Above the tissue and cell, chloroform (1:10 ratio) was added and the mixture was smoothly shaken. After being kept at 25°C for 5 min, the sample was centrifuged at 12,000 rpm for 15 min. The RNA pellet was precipitated with isopropanol and centrifuged at 12,000 rpm for 15 min. After the supernatant was removed, the pellet was washed with 75% ethanol. To remove the ethanol supernatant, the pellet was centrifuged at 12,000 rpm for 15 min and dissolved in diethyl pyrocarbonate-treated water. The RNA (2.0 μg) treated with RNase-free water was reverse-transcribed using a cDNA synthesis master kit (GenDEPOT, Barker, Texas, USA). Quantitative polymerase chain reaction (qPCR) was performed using SYBR green real-time master mix (GeneAll, Seoul, Korea) and a CFX Connect System (Bio-Rad Laboratories, Inc., Hercules, CA, USA). The primer sequences are shown in Tables 1 and 2.

**Table 1:** Rat primer sequences

Rat gene	Sequence
*CYP4A1*-F	ACCTGTTCCAGGCATTGTCA
*CYP4A1*-R	AGAAGGGCAGGAATGAGTGG
*CYP4A14*-F	CTTGATGACACTGGACAC
*CYP4A14*-R	ACTCCATCTGTGTGCTCATG
*ACOX1*-F	TCAGCAGGAGAAATGGATGC
*ACOX1*-R	TGGAAGTTTTCCCAAGTCCC
*CPT1A*-F	AAGCTGTGGCCTTCCAGTTC
*CPT1A*-R	GGATGAAATCACACCCACCA
*SCD1*-F	GTTCTCTGAGACACACGCCG
*SCD1*-R	GGATGAAGCACATGAGCAGG
*FASN*-F	AGTGAGTGTACGGGAGGGCT
*FASN*-R	GCTGGGACACATGTGATGGT
*ACC*-F	GGCACTCTGATCTGGTCACG
*ACC*-R	GCTCCGCACAGATTCTTCAA
*SREBP1c*-F	TGCGAAGTGCTCACAAAAGC
*SREBP1c*-R	ACCACTTCAGGTTTCATGCC
*ChREBP*-F	CCTGAAGACCCAAAGACCAA
*ChREBP*-R	AGATGGAGTGCAGGGCTCTA
*TNFα*-F	ATTGCTCTGTGAGGCGACTG
*TNFα*-R	GGGGCTCTGAGGAGTAGACG
*IL-6*-F	TCATTCTGTCTCGAGCCCAC
*IL-6*-R	GAAGTAGGGAAGGCAGTGGC
*MCP1*-F	GCCAACTCTCACTGAAGCCA
*MCP1*-R	GCATCTGGCTGAGACAGCAC

**Table 2:** Human primer sequences

Human gene	Sequence
*ACOX1*-F	AGGTCACAGCTGTCCAACCA
*ACOX1*-R	TTACCCAGCCCTGGCTTAAT
*CPT1A*-F	GGTCCAGGTAGAGCTCAGGC
*CPT1A*-R	GTGCTCTGAGGCCTTTGTCA
*FASN*-F	GACATCGTCCATTCGTTTGTG
*FASN*-R	CGGATCACCTTCTTGAGCTCC
*ACC*-F	GCTGCTCGGATCACTAGTGAA
*ACC*-R	TTCTGCTATCAGTCTGTCCAG
*SREBP1c*-F	CTCCGGCCACAAGGTACACA
*SREBP1c*-R	GAGGCCCTAAGGGTTGACACAG

### Liver triglyceride measurement

Liver tissue was homogenized in phosphate buffered saline (PBS). Triglycerides were extracted with methanol:chloroform (1:2) at 25°C for 2 h. After eliminating impurities using a filter, the triglyceride liquid solvent was dried. The triglyceride level was evaluated using a kit (Bioassay Systems, Hayward, CA).

### Statistical analysis

All results are expressed as mean ± standard error of the mean (SEM). Treatments were compared using a t-test, followed by a Student's t-distribution. Statistical significance was defined as a *p* value < 0.05. Analyses were performed using GraphPad Prime 5 (GraphPad Software, La Jolla, CA, USA).

## SUPPLEMENTARY FIGURE



## Abbreviations

ACC: acetyl CoA carboxylase
ACOX: peroxisomal acyl-coenzyme A oxidase
AP-1: activator protein-1
ChREBP: carbohydrate-responsive element-binding protein
CPT-1: carnitine palmitoyltransferase I
CYP4A: cytochrome P450 4A
FAS: fatty acid synthase
IL-6: interleukin 6
JNK: c-Jun N-terminal kinase
LXR: liver X receptor
MCP1: monocyte chemoattractant protein 1
NAFLD: non-alcoholic fatty liver disease
NASH: non-alcoholic steatohepatitis
PGC1: PPARγ coactivator 1
RXR: retinoid X receptor
SCD1: stearoyl-CoA desaturase-1
SREBP: sterol regulatory element-binding protein
TNFα: tumor necrosis factor alpha
