# Supplementary material for: Novel PPARα agonist MHY553 alleviates hepatic steatosis by increasing fatty acid oxidation and decreasing inflammation during aging
Source: Oncotarget. 2017 May 8;8(28):46273–85. doi: 10.18632/oncotarget.17695 (PMC5542266; doi:10.18632/oncotarget.17695)
Supplement: Supplementary file 1 [file oncotarget-08-46273-s001.pdf]

Novel PPARα agonist MHY553 alleviates hepatic steatosis by increasing fatty acid oxidation and decreasing inflammation during aging

SUPPLEMENTARY FIGURE

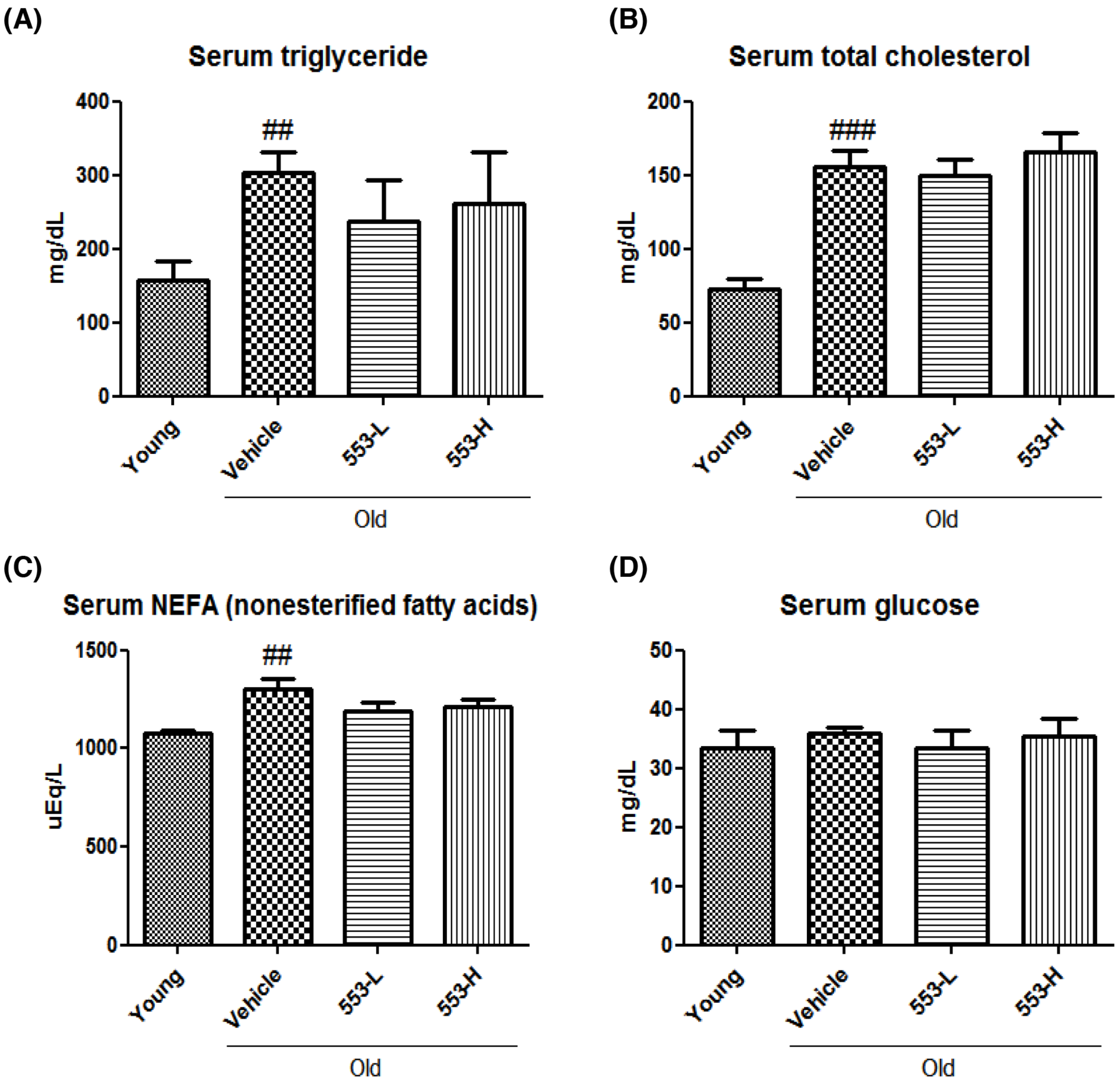

Supplementary Figure 1: MHY553 ameliorates hepatic steatosis level in aging. **(A)** Triglyceride. **(B)** Total cholesterol. **(C)** NEFA. **(D)** Glucose concentration in serum was quantified using the colorimetric assay kit, respectivelyafter administrating MHY553 to aging rats. The data are expressed as a mean ± SEM (n = 6). $^{\#\#}$ $p < 0.01$ *vs*. Young ; ### $p < 0.001$ *vs*. Young.
